# Lectures on Natural and Difficult Parturition

**Published:** 1847-04

**Authors:** 


### PART II.—REVIEWS.

ARTICLE V.

*Lectures on Natural and Difficult Parturition:* By Edward William Murphy, A. M., M. D., Professor of Midwifery, University College, London; Obstetric Physician, University College Hospital; and formerly Assistant Physician to the Dublin Lying-in Hospital. New York: S. S. and Wm. Wood. 1846. pp. 281. (From the Publishers.)

This is a neat little volume, and very convenient, both for the student and practitioner.

The author has wisely refrained from lengthy discussions in reference to the opinions of authors, and confined himself to facts. He has also studied brevity to good advantage, and, in this respect, this little work forms a striking contrast with the large amount of verbose medical literature which has been issuing from the press during the last few years.

The first lecture gives a concise description of the bones of the pelvis, with its measurements, and those of the fœtal head; showing the beautiful mechanism in the adaptation of the diameters of the pelvis to those of the child's head.

The second lecture shows the different modes of measuring the pelvis, and the proportions of its natural form; variations from which constitute deformities. The causes of deformities of the pelvis are briefly discussed, with the use of the different instruments for detecting them; in reference to which he has correctly come to the conclusion, that, where the deformity is great, there is but little use for them, and where it is slight they are not sufficiently accurate to be depended upon. The lecture closes as follows:

"Besides these modes of measuring the pelvis, none of which can be depended upon, there are what have been called 'digital measurements;' or, in other words, the experienced accoucheur, from constant habit, when he passes the fingers or hand into the vagina, will form a very accurate estimate of the space in the pelvis. This is done in different ways: if one or two fingers are pressed towards the promontory of the sacrum, and if they at all approach it, it is certain the promontory projects too much, otherwise this never could happen, as you will find, if you try the experiment on the dried pelvis, sometimes as much of the hand as the vagina will admit, is introduced; if the sacrum below the promontory and pubis are only touched, when the fingers are separated, it indicates sufficient space; if it be impossible to separate them, the contrary; and from the degree to which the fingers are compressed, the amount of disproportion is estimated. In some instances it was impossible to get more than two or even one finger wthin the pelvis."

The third lecture is on the "Mechanism of Parturition," and gives the classification of labors by different authors, and proceeds to the consideration of the manner in which the expulsive forces are produced and applied to the child.

His stages of labor are—1st. The dilatation of the os uteri; 2d. The expulsion of the child; and 3d. The separation and delivery of the placenta—which is the simplest and most natural division.

To show the necessity of not being hasty in rupturing the membranes, a point of great practical importance, we extract the following:

"We would now direct your attention to the means provided by nature to prevent the danger which might arise during this process [dilatation]. If the uterus exerted its full power upon the undilated os uteri, and if the unyielding head of the child were driven forcibly against it, the almost certain consequence would be, that the irritation would excite increased resistance, and ultimately terminate in inflammation of the mouth of the uterus. To obviate such an effect, nature interposes a *fluid medium* between the power and the resistance. The liquor amnii contained within the membranes, occupies the cavity of the uterus, and when its parietes contract upon it, the force exerted is, as we have explained, by this means accurately conveyed to the os uteri. When the latter dilates in the slighest degree, the fluid insinuates within the smallest opening, and expands it by a direct lateral pressure against its edges. The power of the uterus is thus made to act in the most favorable manner for distending its mouth.

Other advantages are also gained. The os uteri may dilate irregularly; but any attempt to overcome forcibly the undilated portion, is prevented when the force is conveyed through a fluid, which, while it readily yields to an undue resistance, still maintains an equable pressure upon the edges of the os uteri. Any irregularity in the action of the uterine fibres, is also, to a certain extent, obviated, because these contractions, although irregular, being still conveyed by the fluid, are thus equally communicated to the os uteri. Further, so long as the tissue of the uterus intervenes, it is necessary to moderate the great power which the uterus is capable of exercising to dilate it: this is effected by the liquor amnii. The force conveyed by a fluid, you are aware, does not act in one direction only, but is distributed to every part of the surface to which the fluid is applied. The force, therefore, which is exerted to expand the mouth of the uterus, being communicated by a fluid, is not only directed against the os tincæ, but against the fundus and sides of that organ. The fundus, consequently, is opposed, not only by the os uteri, but by its own action reflected by the liquor amnii. Hence, so long as the fluid remains and the os uteri is undilated, the more powerful the action of the fundus, the greater is the resistance to it. The actual force employed is, therefore, very moderate, and any sudden or violent effort at distension is altogether obviated. You may observe this in the character of the pains during this stage. You will find, that, however severely they may commence, they last but a short time, and the effect on the os uteri is comparatively slight. If these short, though severe pains, be contrasted with the long continued and powerful pains which follow them when the liquor amnii is discharged, and the os uteri dilated, the difference in effect will be sufficiently obvious. As a means, therefore, of conveying the whole muscular power of the uterus upon the os uteri—of moderating and equalizing the force employed—of dilating the mouth of the uterus without exciting irritation—the liquor amnii is of essential importance."

And he might have added, also, that, where not discharged until full dilitation, at the commencement of the second stage —that of the passage of the child—it renders essential service by lubricating the parts.

In reference to the difficulties often presenting in the first stage, the author observes:

"There are, however, many exceptions to this condition, varying with the degree to which the density of structure in the os uteri may be increased. The cellular tissue is never so loose and permeable in the first instance as it becomes afterwards; the mouth of the uterus is, therefore, more resisting in first than in subsequent pregnancies. Its structure retains more of its elasticity and firmness in young women pregnant for the first time, and consequently much more time is occupied in unfolding it; hence, the first stage of labor is always longer in primiparæ than in those who have had many children. The os uteri is still more firm and resisting, if, in addition to a first pregnancy, the woman be advanced in years: the cervix and os uteri remain close, compact, and impermeable to the moment of parturition, which may be attributed to the increased firmness and diminished vascularity which age produces in the tissues generally. It then obtains the name of *rigid os uteri.* But there are different degrees of rigidity. Sometimes the structure is only *tough.* It gives way very slowly to the action of the uterus—nevertheless, it yields, although, as it were, reluctantly. In such cases, the os uteri may remain cool and free from tenderness, but opposes a firm resistance to the pressure of the finger, and always requires a long time before the dilitation is accomplished. There is, however, a certain class of cases in which this condition of the uterus is in the extreme. It might almost be called the *undilatable os uteri.* In this state its structure is unusually dense, and feels like cartilage. The edge of the os uteri is perfectly unyielding; when thick, it might be compared to the feel of Gimbernat's ligament. If very thin, it still offers the same resistance, and is to the touch like a hole made in parchment. Instances of this extreme rigidity are met with, not only in women who are advanced in life, but in those who have been, all their lives, accustomed to much bodily exertion, and exposed to the vicissitudes of laborious occupations. They are generally hard featured, coarse skinned, muscular women, of low stature, with thick short fingers, large wrists, and the bones generally prominent. It is in these cases you meet with that form of pelvis that I have described to you, as possessing many of the characters of the male pelvis. If you should, unfortunately, meet with a case of this kind, you must be prepared for difficulties from the commencement to the termination of labor, and, therefore, the consideration of it deserves your closest attention.

"All these varieties are included under the term "rigidity." But besides, there are cases where the os uteri becomes *rigid although previously dilatable.* If the os uteri become inflamed, rigidity is the result of it; the os tincæ grows hot and tender, is swollen, and becomes rigid. This alteration may arise from any irritation: premature rupture of the membranes for instance, by which the head is brought into direct contact with the undilated os uteri. It is also often induced, not by accidental causes, but by too much meddling, making too frequent examinations, attempting to dilate the os uteri artificially, etc. You cannot, therefore, be too cautious in this respect. Sometimes the head of the child presses so unequally upon the os uteri as to excite inflammation in it. The head may not be directed exactly in the axis of the brim, but may rather rest upon the pubic portion of it, compressing the anterior lip of the uterus with every pain. While the remaining portion of the mouth of the uterus expands, this remains undilated, and forms a band in front of the head. When the membranes are ruptured, the pressure is so much increased that the anterior lip often inflames and grows quite rigid. Again, there are cases where the os uteri is driven down with the head into the pelvic cavity, and the whole circle of the os tincæ compressed so tightly against the pelvis as to produce inflammation; further dilatation is arrested, the os uteri is rigid, and if it remain long in this condition, slough may be the result; the whole os tincæ has been completely separated in this manner, and expelled with the head of the child."

The fourth lecture is upon the delivery of the child and the placenta—the fifth and sixth on the management of natural labor. The author directs the patient to be placed upon the left side, without mentioning any other position; an omission that might be expected from an English midwife; but, certainly, in those cases where the patient prefers it, and where there is no possible objection, it would be better to allow her to lie upon her back, as is preferred by some of the French authors.

The mode of preparing the patient by placing an oil-cloth or india-rubber next the bed, and cloths over it, may answer in large cities, but such directions will be of little use in the practice of most of our readers. Our author justly condemns the too common practice of patients remaining in their ordinary day apparel during delivery, on the ground that the change of clothes immediately after delivery is extremely hazardous on account of the liability to hæmorrhage, and the fatigue it must produce at a time when repose is most important.

The best plan of preparing the patient for delivery, in this country, is to allow her to clothe herself in her night clothes —those she proposes to wear after delivery—except the skirt, and draw them around the waist under the arms; a thick skirt may then be drawn on, which may be removed after delivery by slipping it down, and another may be put in its place, without requiring the patient to rise up. The night clothes can be drawn down, the bandage applied, and all made comfortable without fatigue or exposure to the patient.

The importance of a proper application of the bandage by the practitioner himself is clearly set forth, and one of an elastic character, as flannel, strongly recommended.

The seventh, eighth, ninth, and tenth lectures are devoted to the consideration of tedious and laborious labors.

The causes of tedious labors are considered under the following heads, viz: over distension of the uterus; extreme obliquity; gradual escape of liquor amnii; hysterical excitement; mental despondency; rigidity of os uteri from inflammation; toughness of os uteri; extreme rigidity, &c. We extract the following:

"5. *Mental Despondency.*—This source of difficulty is but briefly alluded to by the majority of obstetric authors. 'Depressing passions of the mind' are enumerated among the causes that retard labor, but are not dwelt upon in proportion to their importance. Fortunately, extreme cases of this kind are rarely met with; but instances might be quoted in which death was the result of such a cause. Cases are occasionally recorded of *unaccountable sudden death* after labor, which might, perhaps, be explained in this way, if all the circumstances of the case were understood; at least, the few instances that have fallen under my notice, seemed to admit of such an interpretation. In one case death would, undoubtedly, have taken place, had not the cause of depression been so obvious.

"A poor, emaciated woman entered the Dublin Lying-in Hospital, January, 1834, to be delivered of her eighth child. 'Sharp misery had worn her to the bones;' her pulse was feeble, the action of the uterus weak; notwithstanding this, she was delivered in an hour after admission; no hæmorrhage took place, and the placenta was separated without any difficulty, but her delivery was followed by the most alarming depression, which required the utmost care and attention to prevent her sinking altogether. Fortunately, strong beef tea and other nutritious diet, had been given to her from the time of admission, so that, with the addition of stimulants, and maintaining the temperature of the surface, she gradually recovered. This was a case where poverty and starvation produced their usual effects, and consequently one more under the control of treatment than those melancholy instances in which some cause, operating on the mind alone, produces some extreme nervous shock which we cannot relieve, because we cannot 'minister unto a mind diseased.' An instance of this kind occurred in the same institution the following year, January, 1835. A young woman was admitted in labor of her first child. She was evidently above the class of persons usually admitted into that establishment. She seemed rather to shun observation; and there were no symptoms attending labor that required interference. It proceeded to its conclusion without any interruption, and terminated within ten hours from its commencement. The pains were feeble, but they were sufficiently strong for the purpose; the patient herself appeared also weak. She was delivered of a girl; and in about a half an hour after the placenta was expelled; but the pulse instantly sunk, syncope followed, and every means that could be used failed to prevent dissolution, although the discharge from the uterus was not increased, nor was there the least evidence of hæmorrhage, either externally or internally.

"An inspection was made twelve hours after death, and no cause could be discovered to explain an event so unlooked for; her history, however, may do so. She had been one of a respectable family, delicately reared, and educated in the strictest moral principles. She had been seduced, betrayed, and deserted; and, to complete her miseries, had to endure her hour of trial in the reception ward of the Dublin Lying-in Hospital. I shall only mention another instance of this kind, which will, perhaps, more distinctly illustrate the effect of an extreme nervous shock.

"In the beginning of the year 1834, a poor woman had walked some distance to the Dublin Lying-in Hospital, and when near it was suddenly seized with the pains of labor. She was delivered in the street, and with much difficulty brought into the house before the placenta separated. It came away, however, without difficulty; and the trifling hæmorrhage that followed was easily arrested. Her alarm was very great, but after some time it subsided; she slept, and nothing further occurred out of the usual course until the following day. On that morning a patient was brought into the same ward to be delivered who was extremely boisterous; she occupied the next bed to this woman, who lay so quietly that she seemed to pay little attention to the disturbance. In the course of the day, however, she complained of being overcome by her cries. She felt faint as if she were sinking; she had slight pains in the epigastrium, some sickness of stomach, pulse rather rapid, compressible, and soft. The woman who caused this was fortunately delivered, and thus all further annoyance was removed; but this patient did not recover from the effect that it seemed to produce on her. Stimulants were given to her, the extremities and surface kept warm, and the most perfect quietness observed in the ward—but all to no purpose. In the evening she was seized with syncope so alarming as to excite the greatest apprehension for her saftey; the extremities became cold, her motions passed involuntarily, and she died in about three hours. The uterus was perfectly contracted; there was not the slightest appearance of hæmorrhage from the vagina, nor any symptom present to explain the cause of dissolution.

"A very careful inspection was made after death: all the viscera of the abdomen were quite healthy, the uterus firm and contracted to its usual size. There were some old adhesions in the lungs; the heart was small, and contained very little blood on the right side; the vessels were all sound; and the only alteration in the brain was an increased quantity of serum in the ventricles and at the base. No other explanation, therefore, was left, but the probable one, that she sunk in conseqence of extreme nervous shock. Her own sudden delivery produced a strong impression on her mind in the first instance. This was again excited and increased by the violence of the patient alluded to, and hence the effect. It is probable that she would have recovered from the first shock, had it not been again renewed by this accident.

"These instances will illustrate the influence of the mind on the constitution at this critical period; they are, fortunately, rare; but those cases where the same cause operates in retarding, and sometimes in suspending, the action of the uterus, are more frequently met with. The sympathy (to use a popular term) that exists between the brain and the uterus is matter of daily observation, the change of feelings and temper that frequently result from pregnancy, the hallucinations that occur after delivery, from the slightest temporary aberration to long continued mania, all prove the influence of the uterus on the mind. So, on the other hand, a disturbed mind suspends the action of the uterus, just in the same manner as it interferes with the healthy action of the digestive organs. As in the latter class of cases you find the appetite gone, the digestion imperfect, the liver disordered, and the bowels constipated, so in the former parturition may be greatly prolonged, and the patient recovered with difficulty from the effect of a labor that otherwise would have been happily concluded within the average period. Such cases may come under your notice; it is therefore necessary to recollect their characters."

Under the head of laborious labors are considered all those difficulties arising from a want of due proportion in the pelvis or child's head, malposition, tumors, exhaustion, inflammation, &c.

In reference to the use of ergot the author says:

* * * "After a temporary rest has been thus produced, (by opium,) if the uterus still continues to act feebly, ergot of rye may be given in an equally cautious manner, carefully attending to its influence on the pulse, and especially on the circulation of the fœtus. If, in either case, after giving this medicine, the rate of the pulsations be diminished, you must not persevere in its employment, otherwise the death of the child may be the result. It is also necessary to be careful to avoid the use of *secale cornutum*, if the delay in this stage arises from great disproportion between the head and the pelvis. It must be obvious to you, that, in a case like this, it would be very dangerous to use a means of exciting the action of the uterus, over which you can have no control. A preparation which exerts a specific influence on the uterus, which often causes the most violent action, and that not returning at intervals, as ordinary pains do, but which excites a *continuous* effort of the uterus to expel the child, is not the safest to employ when there is much resistance opposed to this action. The remedy, when cautiously administered, is useful, however, in those cases where the delay chiefly arises from want of power in the uterus, which may be exhausted if not thus artificially stimulated to action."

To this is appended a note giving the result of Drs. Beatty and Hardy's experiments with ergot, in which they come to the conclusion that a narcotic effect is produced upon the child in utero by the ergot given to the mother; but it has seemed to me that the deleterious effects upon the child oftener depend upon the constant and forcible contraction of the uterus where delivery is delayed after its administration.

Nothing is said of the use of ergot in preventing hæmorrhage — certainly one of its most important indications. Where the ergot has been given, I have never seen a case where troublesome hæmorrhage followed. I have, therefore, in my lectures, recommended that where there was a known tendency to flooding, or where there was reason to expect it to follow delivery, a dose of the ergot be given a few minutes before the expulsion of the child as a preventative. This practice has been extremely successful in my own hands.

In reference to the propriety of using the forceps in cases of impacted head, our author is certainly an alarmist. Many cases of retarded labor, where the head of the child is confined in the pelvis from slight disproportion or enfeebled action of the uterus, are allowed to remain unassisted until the child is killed, and the mother's parts seriously injured so as to cause contusions, ulcerations, or sloughings to follow, when a resort is had to the forceps just in time for them to be made the scape goat to bear off the sins of this delay.

I have no disposition to make light of the dangers of using the forceps and other instruments—they certainly should be used with the greatest care—but what I do most positively object to is the practical results which follow such alarms in reference to instruments. Fear of their application causing delay until a large part of the benefit to be derived from them is lost. On this account I apprehend there is more of the mischief attributed to instruments dependent than on their awkward application. The author says:

"We are not generally favored with a faithful history of cases that illustrate the mischievous effects produced by the forceps. On the contrary, while the post-partum accidents of a skillful operation are deeply concealed in the shadows of the back ground of the picture, the surprising, the almost miraculous, power of the instrument is put prominently forward, with all the vividness of the most glowing and high colored description. Thus the truth is concealed from you, and so would remain, until exposed by your own dear-bought experience, except that you find scattered through the works of men whose skill is acknowledged, ominous hints and anxious warning against the improper application of these instruments. Many evidences might be quoted to this effect: we shall direct your attention to a few of them. Your late respected professor, Dr. Davis, paid a great deal of attention to the subject of instrumental labors, and was disposed to advocate a much bolder use of the forceps than what I should recommend; nevertheless, he candidly admits, that, 'of all the instruments used in the practice of midwifery, those of the present class [the forceps] are unquestionably *the most dangerous to the mother*, inasmuch as in all cases where the forceps are used, the maternal tissues are more or less liable to contusion. All the fangs and framework are made of tempered steel, and let them be ever so well covered and defended they will still retain a great degree of hardness, calculated to bruise and to fret the soft and living texture which might be interposed between their covered surfaces and the solid walls of the pelvis.'

The same impression of mischief leads Dr. F. Ramsbothom to warn the practitioner that 'cautiously and tenderly must his iron instrument be used! We must recollect that no sensation can be imparted to the the operator's hand of any injury that may be done to the woman; and we must remember that one injudicious thrust, one forcible attempt at introduction, one violent effort at extraction, may bruise, may lacerate, may destroy!' Dr. Blundell addresses his pupils thus —'When, however, you lay your hand upon the tractor or forceps, remember, that the accoucheur who is meddlesome may be guilty of occasioning laceration of the perinæum, rupture of the vagina, compression and death of the child, inflammation of the abdomen of the mother, and many other fatal consequences, *which I myself have had occasion to see*—a list of offences surely sufficient to alarm the prudent.'

"But let us come to more direct evidence. Riecke, in his report of the practice of the kingdom of Wurtemburg, gives the results of a very large number of cases, and amongst them those in which the attempt was made unsuccessfully to remove the impacted head by the forceps. He observes—'Almost always, perforation was preceded by attempts to apply the forceps, and to the great injury of the mothers, because perforations, not preceded by such attempts, presented much more favorable results. * * * * The trials at extractions with the forceps—which many accoucheurs continue to the extinction of the infant's life (although foreseeing the necessity for perforation)—exhaust the mother to that degree that she necessarily sinks under the effect of these violent efforts.' In allusion to similar inquiries, Dr. Collins remarks—'It is from being thoroughly convinced of these facts by long and extensive observation, that I consider the forceps quite inapplicable when the head becomes fixed in the pelvis, and the ear cannot be reached by the finger except by violence, in consequence of disproportion existing between the head and the pelvis. * * * * The results I have witnessed from such practice [delivery by the forceps] were most distressing: in some, the neck of the bladder or urethra either lacerated, or the injury by pressure from forceps so great as to produce sloughing and consequent incontinence of urine; in others, the recto-vaginal septum destroyed, either of which renders the sufferer miserable for life; and in two cases where the mouth of the womb was imperfectly dilated, so much injury was inflicted on this part as to terminate in death.' Dr. R. Lee, in his lectures, quotes the paragraph at full length from which these passages are extracted and adds—'The accuracy of these remarks is fully confirmed by all the forceps cases which have come under my observation, which exceed sixty in number.' It would occupy too much time to accumulate further testimony to the same effect. I trust sufficient has been laid before you to authorize the conclusions at which I have arrived, and which are now submitted to you—viz.: that when the head is impacted in the pelvic cavity, it cannot be delivered by the forceps without such injury to the passages as might endanger the mother's life; that the probability of preserving the child's life is not sufficiently certain to justify an attempt which might be so hazardous; that in the great majority of these cases the death of the child takes place naturally, and it may be removed before symptoms dangerous to the mother present themselves; and, lastly, that if it should happen that the reverse occurs, and danger to the mother—whether from exhaustion or extending inflammation—is indicated before the death of the child, that then perforation is called for, rather than render the risk to the mother a certainty, by the dangers that result from a forcible extraction by the forceps."

Lecture eleventh treats of the use of instruments having in view the preservation of the lives of both mother and child. These instruments are the vectis and forceps. Lecture twelfth is a continuation of the subject of the application of instruments, which is clearly discussed, and the lecture closed by the following judicious remarks on a kindred subject:

"*The induction of premature labor* is one of the greatest improvements in modern practice, because, by its means, the leading principle of obstetric operations may be carried out, and both mother and child preserved, in cases in which otherwise we could hardly hope for such a result, We shall not occupy your time with by-gone discussions on the propriety of prematurely forcing labor; it is sufficient to say, that its propriety—nay, its necessity—is admitted in the cases we have described to you, and the only point to be determined, is the case in which this operation is required, We must recollect that, independently of other objections, we have a strong reason for not inducing premature action of the uterus if it can be avoided. The uterus is not prepared for such a change: the cervix is still unfolding, the connection between the uterus and the placenta is more intimate, the circulation in the uterus less easily diverted into other channels; consequently, you expose your patient to greater risk than at the conclusion of pregnancy, and this you would not be justified in doing without a sufficiently powerful motive. The safety of the child is your justification; but you must have clear proof that it is in danger. You cannot trust to an examination of the pelvis only, because, unless distortion is great, it would be premature to say that the child cannot be delivered. The most certain evidence is the result of previous labors, and in the diseased pelvis you have generally sufficient proof of its necessity. Perforation may have been performed in the previous labor; or, with every successive labor, the contraction of the pelvis may have increased so as to render the last more difficult than that which preceded it. If, in such a case, the previous delivery were completed with much difficulty by the forceps, you may fairly assume that the next will require perforation. Thus, you will generally have sufficient evidence to guide you in these cases; but remember the induction of labor is not suitable in first pregnancies.

Different modes of exciting the action of the uterus have been proposed:—1st. *By direct irritation*, as frictions over this organ, artificial dilatation of the os uteri with the fingers or by the introduction of a sponge tent;—2d. *By the specific action of ergot of rye;*—3d, and lastly, *By deranging the connection between the uterus and the ovum*, either by detaching the membranes from the side of the uterus, or puncturing the membranes and allowing the liquor amnii to escape. Of these means the last is the most certain, but, at the same time, one which it would be preferable to avoid if other means were efficient for the purpose, because the liquor amnii would ensure a more favorable dilatation of the uterus, and the child be more secure. Ergot of rye is unsafe, because of the child, the preservation of which is your only motive for interfering; therefore, artificial dilatation by a sponge tent may be first tried, and if it fail, the membranes may be ruptured with a stilette. The action of the uterus sometimes commences immediately, but it may not begin for twenty-four or forty-eight hours after the operation."

Lecture thirteenth, the last of the series, is devoted to an account of the instruments employed in obstetric practice.

The subjects discussed are illustrated throughout with appropriate cuts.

The volume closes with eighty-four aphorisms that may be useful to the practitioner as setting forth, in a few words, the great principles of the science of obstetrics. We heartily recommend this as one of the least exceptionable works on the subject extant—and in view of its brevity, being such that the busiest practitioner may find time to read it. E.

